# Of not passing: homelessness, addiction, mental health and care during COVID-19

**DOI:** 10.1136/medhum-2021-012367

**Published:** 2022-07-12

**Authors:** Johannes Lenhard, Megan Margetts, Eana Meng

**Affiliations:** 1 University of Cambridge, Cambridge, Cambridgeshire, UK; 2 Harvard Medical School, Boston, Massachusetts, USA

**Keywords:** Anthropology, COVID-19, Drug and alcohol misuse, Mental health care, Health policy

## Abstract

People experiencing homelessness in the UK were unconditionally offered housing (and support) from the beginning of the first lockdown in March 2020. For many, that meant ‘(re)entering’ the support system and having a chance to ‘move on’ to longer-term housing. This beneficial effect of some of the policy reactions to the pandemic on people experiencing homelessness was unexpected. On the flip side, however, particularly for people struggling with drug use and mental health issues, adequate support was not available for long periods of time; support was either suspended temporarily or people were excluded from institutional support for not adhering to, for instance, lockdown rules. Similarly, digital support alternatives—modelled on increasingly widespread telemedicine—did often not work specifically for people struggling with complex needs or women experiencing homelessness. This research paper reports detailed evidence of what we observed as continued and catalysed exclusions based on interviews and ethnographic observations with both people experiencing homelessness and service providers from the beginning of the COVID-19 pandemic. Referring to our insights and learnings from three locally and temporally overlapping research projects between May 2020 and April 2021, we also propose changes to redesign future (health)care provision to prevent such impasses—which extend beyond lockdown situations to general conditional housing and support.

## COVID-19 homelessness and care

While many groups: elderly people (Chen *et al*. 2021), communities of colour and people with disabilities (Abrams and Szefler 2020; Dovie, Mariela, and Magdalena 2020) were hit especially hard by the pandemic (and the effects of lockdowns, in particular) forcing many to lose their jobs and potentially their homes, people experiencing homelessness experienced a silver lining during COVID-19 in the form of UK government funding (Ian Hamilton: Covid-19—Are We Rationing Who We Care about? - *The BMJ*). Financed mostly by the Treasury (“£105 Million to Keep Rough Sleepers Safe and off the Streets during Coronavirus Pandemic - GOV.UK.”) under an extension of the ‘Homelessness Initiative’ of 2018, the scheme entitled ‘Everyone in’ (Crisis 2020), was focused on providing emergency accommodation during the lockdown as well as the promise to create 3300 move on homes by the end of March 2021 (COVID-19: Housing People Sleeping Rough - Public Accounts Committee - House of Commons).

While effective for some—with 37 000 people given emergency accommodation (‘Everyone In” Scheme Exposed Gaps in Government, Crisis UK)—the needs of some groups, particularly those requiring specialist healthcare services, continued to be overlooked (Homeless Migrants Still Sleeping Rough despite PM’s Pledge). Based on almost 1 year of ethnographic fieldwork and interviews, performed by the three authors in joint and individual projects in a UK university town, in this paper, we focus on two particular kinds of exclusion. Abstracting from observations from a multitude of support institutions for people experiencing homelessness (emergency housing/assessment centre, Covid-specific hotel, healthcare providers), we illuminate both the exclusion of people experiencing homelessness with complex needs and problems surrounding digital support, specifically for women in situations of homelessness. While these exclusions are not new or *caused* by COVID-19 and the surrounding lockdown measures, the measures made the exclusion of some people particularly visible. On the one hand, additional rules (eg, during lockdown) led to the exclusion to specific groups (eg, people with complex needs) to protect the majority, for example, in a Covid-hotel; on the other hand, shifting service provision (from inperson to online) also affected certain groups (eg, women experiencing homelessness). All of this happened *despite* additional funding available—which itself still had a positive impact for many people experiencing homelessness. We believe our observations show issues on a systemic level with the care provision for people experiencing homelessness which has—over years—become atomised and disadvantaged people who are already more marginalised. Consequently, in the final section of the paper, we propose several (counter) measures to set up a more inclusive system of care provisions.

## Methods and participant involvement

Applying rapid ethnographic assessment (Sangaramoorthy and Kroeger) with a focus on listening to different stakeholders in the community (Listening Carefully to Communities Should Inform Our Response to Covid-19 - *The BMJ*), the three coauthors conducted several months of anthropological fieldwork and semistructured interviews adhering to COVID-19 restrictions in a variety of homeless support institutions in a UK university town between May 2020 and April 2021. Between the three authors, we conducted several dozen interviews with people working in social services (over 30 interviews with more than 15 people) and people experiencing homelessness (9 formal interviews; around 20 informal conversations; with people in different kinds of supported accommodation at the time of the interviews). All institutions—anonymised in this paper—were informed about the nature of this research and agreed to participate (through consent from senior executives/managers as well as employees) and so were all individual participants (whose names are also all anonymised). For each interview with a person experiencing homelessness (seven people) we made it a specific priority of soliciting verbal (and where situationally possible, written) consent—based on detailed explanations of the exact nature and purpose of the research. Informants were not compensated for their participation.

Two of the three core research projects (led by EM and MM, guided by JL as Principle Investigator (PI)) underlying this paper were funded by a rapid response grant as well as a later-stage impact grant at the University of Cambridge (and the Economic and Social Research Council), specifically targeting COVID-19-related projects; the third project (led by MM under the guidance of JL as the PI) was unfunded and conducted in cooperation with several local homeless service providers. The first project was focused on tracing the impact of COVID-19 and the first lockdown on people experiencing homelessness; it involved ethnographic and interview-based research in an emergency housing provider and lasted from May 2020 until November 2020. The second project followed the first in time (November 2020 to May 2021) and focus (engaging local stakeholders to observe the impact of COVID-19 on people experiencing homelessness); it led to the establishment of the Cambridge Homelessness Impact and Research Network (CHIRN) in April 2021. The third project, facilitated by the newly established research exchange by CHIRN, started in December 2020 and will be closed by the end of Q1 2022 with a final assessment report and stakeholder workshop; it was focused on an experiment trialling telemedical means to enable easier access to healthcare for people experiencing homelessness while in a local Covid-hotel between January and May 2021. This project, like the first, involved stakeholder interviews and several days of ethnographic observation in the Covid-hotel and adjacent service providing institutions. The three research projects hence overall provide a comprehensive overview of the local context of service provision for people experiencing homelessness between different kinds of housing and healthcare; the people experiencing homelessness circling and passing through (sometimes for years, as confirmed by senior staff of service providers) the different service and housing providers are connecting the institutions in a deeply embodied way.

The research questions and design as detailed above were informed originally by the insights from several of the support practitioners involved in institutions where fieldwork was conducted; several consultations with the practitioners at key stages of the project helped further shape the directions of research as well as its analysis. The data we processed for this article consisted of interview transcripts from all three projects (with both service providers and people experiencing homelessness; written up and edited for clarity based on initial Otter.AI transcription) and notes from fieldwork encounters (from JL and EM). According to anthropological and ethnographic convention (Mol 2008; Sangaramoorthy and Kroeger, n.d.) the data was not formally coded but organised in case-study-like narratives (mostly centred either around individual people or specific situations, based on ‘thick description’) (Geertz and Darnton 2017), individual vignettes and specific fragments (Veer and Thomas 2016). Comparing both findings from our different but connected field sites and individual analysis from the three coauthors led to our initial set of insights and the establishment of exclusion as a core vector (Candea 2018; Schnegg and Lowe 2020). All three coauthors participated in the analysis leading initially to the production of short reports based on our preliminary findings per research project. In early 2021, we consulted with and presented these preliminary findings to a (local) institutional group of people with lived experience; their feedback and further questions shaped both the last phase of research and analysis and our recommendations (see final section below). We will disseminate the results of the research leading to this paper through our established research-exchange community (CHIRN), recently founded by two of the authors, which includes service providers and people with lived experience and is open to the interested public. All three projects passed ethical review in the University of Cambridge’s anthropology department. No clinical settings were part of the study so no other specific approval was required. Moreover, all relevant (institutional) parties mentioned above were already consulted individually with the findings of this research and were able to provide feedback.

## Multiple exclusions: substance use, complex needs and digital shortcomings

As care provision for people experiencing homelessness, including healthcare, has become increasingly more specialised, it has been divided into individual systems rather than focused on providing a holistic approach. For the general population, care similarly often has become disjointed and uncoordinated with each patient seeing several doctors to provide adequate care for one condition. For people experiencing homelessness, the support they are (often) dependent on has been further under pressure. Generally, driven by de-institutionalisation and a push for community care as early as the 1970s in the UK (Baldwin 1993; Craig and Timms 2009) and more recently austerity measures and welfare reforms, service provision has become reduced and fragmented for people experiencing homelessness (Cameron *et al*. 2016; Gunner *et al*. 2019). In the local set-up where our research studies took place, we observed this development acutely already pre-COVID-19: different parts of support and care provision for people experiencing homelessness were distributed both geographically and institutionally. Specialist, individual service providers were respectively responsible for the provision of housing, benefits, education/training, substance use (including support groups), drug testing, mental health support (if available at all) and physical health. Any individual person experiencing homelessness and struggling with a variety of issues would be tasked to interact with as many different service providers as that person was encountering problems. In few cases, support workers (eg, at the place of housing provision) were tasked with and able to provide coordination and continuity of care. In fact, the commissioning of the different pieces of the support network was done by different parts of the local councils, to begin with (drug use, housing, healthcare—generally all treated separately) going back to individual budgets (per issue area). As a result, people experiencing homelessness in general were—driven by large institutional changes over decades—forced to operate a multistakeholder, specialised and fragmented service provision system; in our specific empirical context, they were only occasionally supported and accompanied through the entirety of this system by a specific support worker. Additionally, we observed a lack of institutionalised coordination and even communication (eg, supported by a single software system) by the support providers making the desired continuity of care and support (Jego *et al*. 2016; Lamanna *et al*. 2018) complicated if not impossible and leaving many people experiencing homelessness without access to the care they needed.

For people experiencing homelessness and struggling with complex needs, navigating a fragmented healthcare system is even more challenging. The National Institute for Health and Care Excellence (NICE) defines people with ‘complex needs’ as ‘people needing a high level of support with many aspects of their daily life and relying on a range of health and social care services’ either because of ‘illness, disability, broader life circumstances or a combination of these’ (Guideline Scope Adults with Complex Needs: Social Work Interventions Including Assessment, Care Management and Support). So not only do people with complex needs have more support needs (eg, housing as well as mental health and substance use support), they also struggle more likely with navigating everyday administrative tasks (Viertiö *et al*. 2012; Dewa, Thompson, and Jacobs 2011). Overall, it is well established that individuals experiencing homelessness are at a greater risk of negative health and social outcomes, with an average life expectancy of 45 years for men and 43 years for women (30–40 years less than the UK national average) (Deaths of Homeless People in England and Wales - Office for National Statistics). Unsurprisingly, numerous studies have highlighted disproportionate levels of cardiac problems, long-standing tuberculosis, hepatitis C compared with age-matched housed individuals (Aldridge *et al*. 2018). This trend is also seen in mental health: a recent study by Groundswell found that 64% of homeless women questioned were experiencing mental health problems compared with 21% within the general population (About Groundswell 2020).

People experiencing homelessness and struggling with specific and often complex needs often require support from multiple specialists to manage their care; many of these individuals would also need additional support from a care coordinator to oversee appointments and advocate for their needs.However, currently, there is very little research into how to best support people with complex care needs (Fortin *et a*l. 2005; Fortin *et al.* 2007) leaving many people to fall between the cracks (Coleman 2003) of support. It was only in October 2021 that NICE compiled its first-ever draft guideline for the ‘Integrated health and care for people experiencing homelessness’ (NICE 2021); so far, many clinicians are unaware of this new guidance and do not use it routinely in their clinical practice. Although a handful of specialist homeless mental health services do exist, most have been disbanded due to lack of funding (St Mungo’s 2011).

While this exclusion is partly driven by the fragmented set-up of the support provision system, an additional factor is discrimination (Homeless People Are Among the Most Vulnerable to the Coronavirus. Yale Psychiatry’s Lo Is Making Sure They Still Receive Care Amid the Pandemic. < Yale School of Medicine), and yet another factor is strict exclusion criteria for certain services, for example, around the so-called condition of being housing- or treatment-ready (Padgett *et al*. 2010; Padgett, Henwood, and Tsemberis 2015). As a result, an additional burden falls hardest on those with complex needs or dual diagnosis (concurrent mental health problems and substance use) (Schütz *et al*. 2019), where (despite regulation changes) these individuals are continuously sent from service to service as they do not meet the ‘treatment-ready’ requirements for substance use or stable mental health, (St Mungo’s 2011).

With the onset of COVID-19, the already atomised and hard-to-access resources were further stretched and access made complicated (Shock to the System: COVID-19’s Long-Term Impact on the NHS - The Health Foundation; Aburto *et al*. 2021; Propper, Isabel Stockton, and Stoye). In the empirical context of our research studies, we observed how service provision for people experiencing homelessness were either reduced in scope (eg, fewer beds offered to adhere to social distancing, certain services (like drug testing) suspended for the same reasons) or altogether (temporarily) suspended (eg, drug and substance use support groups were suspended for several months during the first lockdown in our local context). Additionally, given the lack of overall specific government instructions for homeless support institutions at the beginning of the lockdowns, guidelines had to be made up on the spot (eg, by the management of service providers) and were often changing and unclear; eventually, these guidelines became stricter as we explain below, particularly impacting people with specific support needs to protect what was perceived to be the (more compliant) majority of people. Individuals experiencing homelessness were on the one hand supported more, particularly with housing, with additional government funding being made available. On the other hand, however, specialised support (eg, for people with complex needs or dual diagnosis) was even harder to access. This was also not alleviated for certain groups (eg, women) with a (experimental) shift to digital platforms as we will show further down below. With a lack of suitable technology and inadequate training many of these individuals were and continue to be unable to access even basic (health) services—often both digitally and inperson—which increases their reliance on underfunded but trusted non-profit organisations to fill in the gaps.

## Addiction hurts, at least twice, during COVID

Ben really isn’t keeping up well. He looks fine, but he’s been complaining and he’s constantly out. […] You don’t see a change with everyone, but with him […] it’s so obvious. He is back on the crack, too. And he was supposed to move out soon – but at the moment nobody is moving. They are all staying longer than the 28 days [the supposed length of an average stay at the assessment centre].

Dannie explained to me (JL) who Ben was while we were standing behind the kitchen counter during an evening which I spent volunteering at a short-term hostel (the Centre) in a UK university town in April 2020. Dannie was a long-term volunteer and familiar with many of the people circling through the centre and my partner on that particular evening. Ben was obviously not doing very well; he was zigzagging through the room towards the counter we stood behind, trying not to fall. Dannie and I were responsible for serving the dinner—one of the tasks for the two evening volunteers—and Ben had come to get his. While COVID-19 restrictions, such as social distancing, were taken very seriously in the Centre, there wasn’t enough space for that during dinner. As a result, residents were sitting on the couches; a loud action movie was running on the television. When Ben arrived at the counter, we both smiled at him: ‘Do you also want dinner? Any special requests?’. Looking up briefly, Ben’s teeth were moving and he fumbled around with his hands in his pockets; he wasn’t able to stand still. Mumbling a short ‘No’, he quickly grabbed his plate and disappeared within minutes, having gulped down his dinner.

At the beginning of the full restrictions of the first COVID-19-related lockdown in the UK, homeless service providers were facing their limits and so were the people depending on their support and care. In a *Harvard Health* post from 2020, a US-front-line doctor (and recovered opiate user) contextualises the double hit we observed also in the UK (A Tale of Two Epidemics: When COVID-19 and Opioid Addiction Collide - Harvard Health). Reporting on what he calls the ‘two great epidemics of our generation’, he argues that people suffering from substance use on the one hand ‘are vastly more vulnerable to coronavirus’; on the other hand, they also suffer from shortages of supplies (of methadone and other medications, for instance, at times even clean needles) and of increased issues of isolation, specifically when it comes to the lack of access to their recovery community and peer-support groups that were so often suspended during the pandemic lockdowns.

In the UK context, we observed both of these issues hitting people like Ben hard, too. Ben was young, reasonably healthy and very much used to accessing his friendship group—some of whom were not experiencing homelessness—outside the Centre most hours of most days before the pandemic and its restrictions. His friendship group also doubled as his circle of drug buddies, people Ben would consume drugs with every day, as well as pool money with to buy supplies more cheaply. While his drug use—as he explained in an interview later, of crack and cocaine—continued before the pandemic, Ben was seen as doing reasonably well; he was on track to access longer-term housing, as one of the assessment centre’s managers explained in a later conversation. During the first lockdown, as institutions such as the Centre were experimenting with how to best protect their community, leaving the Centre was not permitted, apart from for specific (mostly medical and ‘exercise-related’) reasons. As a result, Ben was not regularly allowed to go out and hence not able to access the community he usually relied on (‘I rely a lot on my community […] experienced a lot of community through prison’, he explained in an interview with JL in summer 2020). While he breached this rule for a while on an almost daily basis (and was initially not sanctioned), his drug use was rapidly increasing; Ben called it ‘a sort of non-stop relapse’ in the interview with JL, including smoking crack in the assessment centre (‘one of the times the fire alarm went off’). Difficulties with accessing drug supplies (drug dealers weren’t out as frequently and buyers had to travel farther) made his drug use more complicated and forced him to go outside frequently and for long stretches of time, against the rules eventually installed and enforced by the Centre. Given an explanation from one of the support workers familiar with Ben (and the recorded history this support worker mentioned), we can assume that he was additionally struggling with mental health issues, such as anxiety and depression (while he did not mention those in the interview with us). A vicious circle was pushing Ben to the point where JL first encountered him (see initial vignette above).

Even though staff and volunteers at the Centre noticed the increased support needs for Ben and others (as our interviews showed explicitly), there was very little specific support they could offer, particularly during the first lockdown in spring/summer 2020. Ben, for instance, had relied on regular inhouse drug testing to keep himself accountable (‘to rebuild (his) life’, as he explained); that support stopped to adhere to social distancing and as a precaution to keep staff safe. Internally, a major concern was to protect staff (from COVID-19 infections) in the different institutions we observed to keep a minimum of operations functional. Already before the pandemic, the vast majority of specialised mental health and substance use support services had been outsourced systematically to specific service providers; the local substance use support service stopped providing any support during the first lockdown and switched to phone support eventually after a several-months long suspension.

At the Centre, one way of coping with the increased support need and burden was to experiment with changing rules. While COVID-19-related lockdown rules (as mandated in other institutional contexts by the UK government) were introduced, other rules were relaxed. One of the Centre managers, John, explained how balancing residents’ needs and the responsibility to protect staff (and the functioning of the Centre as a whole) was complicated but something they attempted by granting residents certain liberties inhouse. While the Centre—like most service providers—was a ‘dry Covid-hotel’, that is, no alcohol (or other substances) was allowed on-site, a specific area downstairs was opened up for the consumption of certain (weak, up to 5% vol) alcohol during the first lockdown in April 2020. This rule was further relaxed—removing any restrictions on the type of alcohol allowed—several weeks into the lockdown. But people’s rooms were out of bounds, still. John reflected: ‘How are we supposed to keep that [being a dry Covid-hotel] up when people aren’t allowed to leave? They aren’t even allowed to sit in the park. […] Many of our residents have a substance use problems. […] That’s the least I can do. We even buy it for people if they are self-isolating and ask us to. […] One of our biggest problems is keeping people inside, behavioural issues, really’. While for many residents—in the Centre and many other homeless institutions—relaxing restrictions around alcohol consumption were tremendously helpful in a time of stress and crisis, as the case of Ben above shows other substances were even more complicated to manage.

While restrictions around consuming ‘hard drugs’ were impossible to relax legally, Centre staff showed a certain kind of lenience with Ben and others initially. Another staff member, Ollie, explained to JL in an interview: ‘I know from personal experience, if you are in (drug or alcohol) withdrawal, nothing will stop you going out to get your substance; coronavirus won’t even cross your mind’. Ollie was sympathetic with the struggle of people like Ben, but also wary of handing over a blank check to people who use drugs in a situation of crisis: ‘If you turn a blind eye to it: drug-dealing, drug-sharing, violence, reliance on each other – it’s a whole different culture!’. The staff seemed to be united in being unable to alleviate Ben’s particular struggle—fearing the situation could slide completely out of control. Setting a wrong precedent was deemed even more dangerous than supporting people—in whatever way or form was possible—under exceptional circumstances.

Over time, with lockdown restrictions staying in place for months, management at the Centre was increasingly overwhelmed; while Ben’s problems were significant, others were struggling, too. More and more staff were self-isolating, putting additional pressure on the remaining members of the team. Managing the day-to-day operations became a stretch, to begin with, and the team overall managed incredibly well (there was not a single case of COVID-19 reported among residents in the assessment centre in 2020). Dealing with people who had increased support needs was putting a lot of pressure on the functioning of the institution as a whole. The immediate goal for management was to enable *all* residents to be as supported as possible; they were facing a trade-off as Ben and others would not stop excursions outside and put all people in danger (of ‘importing’ COVID-19 into the Centre). Ben himself reflected that he was surprised to be ‘let loose’ for so long: ‘(I had) so many final warnings. […] they let us go four or five (hours) […] then they finally cracked down’. Faced with a decision to be lenient with some or keeping the majority of the Centre (and its staff) as secure as possible, management ultimately decided to stay on the side of more solid enforcement of rules and structure. Over the course of several weeks in late spring 2020, not only Ben but several other people—most of them struggling with complex needs between mental health and substance use—were evicted (or as I was told: ‘asked to leave’) from the Centre after receiving at least two warnings but continued to breach the ‘stay at home’ order.

At the Centre in the UK university town where we conducted a significant portion of our research, but also in many other homeless service institutions, emergency shelters and temporary ‘COVID-19 hotels’ in the UK, Europe and the USA (Homeless People Are Among the Most Vulnerable to the Coronavirus. Yale Psychiatry’s Lo Is Making Sure They Still Receive Care Amid the Pandemic. < Yale School of Medicine; Homeless in Europe the impact of COVID-19 on Homeless people and services contents) the people who were hit most by COVID-19 and the reactions to it were among the most marginalised and in need. Given that most specialised support—particularly for people who use drugs and/or struggle with mental health issues—has historically become outsourced to specific service providers (including general practitioners (GPs)), restrictions of the free movement of people hit those particularly hard who were dependent on accessing a larger variety of different support providers. Many were not able to access any support for their often ever-increasing support needs during the pandemic. Even when some support shifted online or to the telephone (often until today, see below), people with complex needs continued to struggle to engage. COVID-19 (and the restrictions around it) hit people with complex needs at least twice: while stress levels and mental health needs increased (eg, due to isolation, fear of catching COVID-19, no ability to meet friends, new behavioural rules (particularly during periods of lockdown), self-medication with drugs was made more complicated. Not only was supply harder to come by on the street (and so was money from begging, often needed as supplementary income), people in places like the Centre were also simply not supposed to be outside, a rule that was increasingly enforced. Lockdown not only disrupted normal routines, but it also quasi-criminalised some of the routine behaviours our informants’ well-being depended on, either by government-imposed or institutional rules (eg, ‘hanging out’ with friends outside). As a result of the ‘misbehaviour’ and ‘disobedience’, some residents like Ben were eventually evicted from the support institutions, to protect ‘the majority inside’. As a final result, being back on the street, left people (at least temporarily) even further marginalised from healthcare support (beyond street outreach which laudably was in operation throughout most of the lockdowns).

## Digital care? talk of the future…

From the outset, a shift to digital consultations promises to change the landscape of healthcare access to the better, also for people experiencing homelessness.

People no longer need to travel to appointments or leave their places of residence or comfort (eg, a day centre); digital technology allows them to access healthcare services from anywhere with the right mobile device and sufficient signal (You Mean I Don’t Have to Show Up? The Promise of Telemedicine - The New York Times).

While the use of video consultations in healthcare has steadily been increasing for a number of years (Car *et al*. 2020), it was with the start of the COVID-19 pandemic that we have seen another sudden shift to remote consultation, in some settings completely replacing any face-to-face interaction. To protect both patients and doctors, inperson consultations were reduced to a minimum across the UK’s healthcare system already during the first lockdown. Telephone triage became the new normal and doctors and patients alike were forced to learn to adapt to new methods of communication, including specific applications (eg, accuRx (General Practice)).

Although mobile phone use and texting are very common among people experiencing homelessness (with between 89% and 94% of individuals having regular access to a mobile device (Moczygemba *et al*. 2017)), many of the mobile devices provided are unable to support video consultations and those that do tend to be unaffordable (Moczygemba *et al*. 2017). As a result, with more (healthcare) services shifting to digital platforms during COVID-19, the chasm of digital inequalities continued to widen: 79% of homeless service providers screened reported increasing issues due to lack of digital access for people experiencing homelessness in the first lockdown (Boobis and Albanese 2020).

In an attempt to overcome some of the barriers associated with a lack of suitable technology and inaccessibility, a GP service in an unnamed UK university town trialled the provision of ‘mobile health consulting’ specifically with one so-called temporary ‘COVID-19 hotel’ for people experiencing homelessness. The set-up was simple: use (video) telemedicine to connect people experiencing homelessness in a local Covid-hotel directly (and quasi on-demand) to a specialised GP service for non-emergency healthcare consultations.

The implementation of this new service divided the opinions of both staff and the people who used it in the COVID-19 hotel. While some staff—doctors and nurses alike—fundamentally did not agree with the idea of video consultations as a substitution for (or even addition to) inperson consultations, other staff wholeheartedly supported the idea. Several staff members commented that with the introduction of video consultations, they saw ‘terrific engagement from many clients who would not normally engage with the service’. One key success story was with Brody, one of the residents of the local COVID-19 hotel:

Brody was a deeply entrenched individual; encouraging this person to go to the surgery was always a difficult task […] suddenly, we were able to provide a consultation which meant that all we had to do was go from one side of the building to the other. It was a real success to get him involved. Support worker

The benefit of new and unseen engagement was particularly noted in people with limited mobility. As two people with lived experience noted, not having to travel to see a doctor also made it easier for people who struggle with anxiety or depression (and might not want to leave their house) to connect to healthcare in an environment they felt comfortable in. One person who used the service commented:

While the video appointment was still daunting it was far less scary than a face to face appointment and definitely helped with my anxiety around seeing health professionals

For the healthcare providers, video consultation can also solve several issues at once: they make DNA (people who ‘do not attend’ the appointment) less likely, ensuring the continuing provision of consultation also during lockdown conditions while reducing (inperson) appointments (potentially dangerous for vulnerable people). For one nurse frequently in contact with people experiencing homelessness during street outreach tours, this was a very strong point: *‘It is another string to my bow that I can offer people when I am out on outreach’.*

However, from our time-constrained small-scale observations during the one local trial we accompanied, only a certain group of people, a subgroup of people experiencing homelessness, were able to enjoy the benefits. The ‘success stories’ of video consultations we observed were with young male individuals who were already familiar with video communication. Women, older individuals and those who were less comfortable with technology remained less or not at all engaged. This highlights several problems with the service more generally and some general principles of how to design (and test) similar services in the future.

### Privacy needed, particularly for women

During the 3 months of this particular study at the local Covid-hotel, no women requested a video consultation and one female person living in the hotel which MM encountered during fieldwork asked specifically to speak inperson to a GP as she was concerned about privacy. The idea of speaking openly about physical and mental health problems is often challenging (Gangi *et al*. 2016; Mak and Wu 2006). Fear, stigma, relationship to the listener and the perception that mental health problems are less important than physical health are all sighted as reasons that contribute to a delayed presentation of people with mental health problems and trauma to primary and secondary care (Grice 2016; Reavley, Morgan, and Jorm 2018; Bedard-Gilligan *et al*. 2012; Bolton 2003; (US), Center for Substance Abuse Treatment 2014).

Hence, while the consultations were held in a private space or the person’s room (depending on their choice), there was still fear that other residents could overhear the conversation or even hear that a consultation happened. Furthermore, with no referral pathway that was anonymous or allowed the person requesting the consultation to contact the GP directly to make an appointment, people were required to speak to a member of staff both to make the original referral and to get access to the equipment for the consultation. Finally, a lack of client training sessions meant that digital privacy was not addressed and individuals who were unfamiliar with the technology often required facilitation (by members of staff) during the consultations which again may have made it more difficult for some individuals to open up.1 This might have been another reason for some individuals within the hotel not to access the service. However, from interviewing GPs working within the sector, the lack of engagement of women experiencing homelessness in healthcare was seen to be a systemic problem. This is also reflected within a recent study by Groundswell which highlighted that 65% of homeless women interviewed struggled to find the motivation and confidence to deal with health issues (Groundswell). This highlights that a lot more research needs to be done to look into why homeless women are not using primary care services and what we can do to improve this, particularly with a focus on telemedicine which is projected to become increasingly widespread.

### Technological barriers

The greatest concerns among participants, healthcare professionals and people in the co-production group we consulted were around technological barriers. How could you ensure the availability of a good enough (phone) connection, good camera resolution, strong enough phone volume and most importantly the technological literacy required to use such a mobile device generally? This point was particularly evident during one consultation with an elderly resident in the hotel. The poor sound quality meant that the resident needed to repeat parts of his story two or three times; this led to frustration and made it incredibly difficult to build up a rapport between the doctor and the person. This frustration was furthered by the small screen size which limited the doctor’s view and made it more difficult to assess how the person was coping. As one nurse stated:

A lot of information in a face to face consultation […] cannot be caught on camera, particularly the way the person walks into the room. For example, drug-seeking people may be dancing out the front of the building but when they come in they may be asking for pregabalin for their pain

All of these issues were encountered during the trial phase despite the relative stability of the Wi-Fi connection in the COVID-19 hotel. Taking the technology of video consulting to a different context (eg, people experiencing homelessness calling in themselves, possibly from a street setting) risks increasing the frequency of the technological problems drastically.

While several studies have suggested that people experiencing homelessness are willing to engage in digital forms of support for managing their mental health and prescriptions (Jennings *et al*. 2016; Glover *et al*. 2019), the digital divide must be addressed before telemedicine technology can be considered to be a feasible method for improving healthcare accessibility among individuals experiencing homelessness.

While phone access is widespread (Rhoades *et al*. 2017; McInnes *et al*. 2014), only 50% of the devices could support video calls. Furthermore, maintaining telephone connectivity is often challenging due to financial constraints. (Jennings *et al*. 2016), hence additional funding would be required to ensure that all individuals had access to reliable technology.

Although ensuring adequate privacy and overcoming technological problems were both barriers that contributed to the limited success and uptake of the service, the sudden set-up of the service and the failures to involve all relevant parties also might have contributed to the difficulties we observed.

First, not all GPs at the practice were engaged with the telemedicine project. This meant that people who requested a video consultation would often be placed with an unfamiliar GP practitioner. For a consultation to be successful there must be a strong trusting relationship between the doctor and patient (Ruberton *et al.* 2016; Skirbekk *et al*. 2011; Grassi, Caruso, and Costantini 2015); this is even more important for individuals experiencing homelessness, many of whom have a history of trauma and negative previous experiences of healthcare services. One person, Tim, mentioned this problem explicitly. Although support workers had commented that it had been a *‘great success’* to get Tim involved in a consultation, he was both unsettled by the new technology and was far less willing to engage and open up to an unfamiliar healthcare professional. It was only after the telemedical consultation that Tim was visited inperson by his regular GP; during that visit, Tim received what the doctor afterwards called ‘satisfactory care’. Tim even commented that he may be willing to engage in a telephone consultation (despite his preferences for face-to-face visits) as long as it was with his regular doctor. This experience reflects the view of many healthcare workers we interviewed, who commented that it is far more successful to build rapport in a face-to-face context with a new person before moving to a digital platform in a second step.

The second problem resulted from a lack of more general preparation and set-up of the service, particularly in terms of missing staff training and not raising enough awareness among hotel residents. Publicity for the digital consulting problem was primarily through word of mouth which in itself introduced unconscious bias and may have meant that not all residents were made aware of the service. It was only at the end of the project that information leaflets were created to inform people in the Covid-hotel about the service. To add to the confusion, healthcare staff from the GP service (outside the core group of doctors and nurses) and within the Covid-hotel were either unaware of the service or unclear about which people should be referred in what way. Both of these faults could partially explain the general lack of uptake from people during the project, with less than eight video consultations performed over 3 months, most of which fell in the last month. Better, more direct and more comprehensive communication from the beginning of the roll-out period would likely alleviate this problem.

In summary, the first-of-its-kind local experiment to introduce telemedicine to promote inclusivity and widen access to healthcare for people experiencing homelessness had mixed results; the rapid implementation of the project (without, eg, adequate accompanying communication and onboarding for all relevant parties) could be seen as ‘teething issues’. But the trial we observed also highlighted more general problems of adequate privacy and technological barriers likely to be aggravated in less ideal set-ups. On some level the service did fulfil its role in allowing a small proportion of individuals to access healthcare who otherwise would not engage, however in doing so, it ostracised others from accessing the services they required; in particular, we observed this happened to women who experienced homelessness. Moving forward the first experiences of providing telemedicine to people experiencing homelessness did have positive outcomes which could be applied to other services (eg, mental health provision (Schueller *et al*. 2019), or mobile-based buprenorphine treatment (Iheanacho, Payne, and Tsai 2020)) but this will only be effective if we take onboard the lessons we learnt from this pilot study. We will go into some more depth with our recommendations in the following, final section.

### Learnings: provide more specialised care and accelerate the right digital services towards an integrated system of care

Based on our key findings around the impacts of COVID-19 and the accompanying responses in the system of service provision for people experiencing homelessness, we propose three tangible suggestions to improve the delivery of such services; we imagine that a system of care and support is possible which is specialised but integrated and can harness the potential of telemedicine to widen access for people experiencing homelessness, especially to healthcare.

1. *Particular attention and specialised care for those struggling with substance use and mental health*. By far, those managing substance use, mental health conditions or both (complex needs/dual diagnosis) were most strongly and negatively impacted by the pandemic and the mitigation strategies around it. The safety net was not sturdy enough—or too atomised—to catch, in particular, people with more complex needs. As such, money and resources should go to specialised services for complex needs (ie, hiring support workers with training in dual diagnoses). While people we spoke with and observed suffered particularly from the temporary closure of some specialised services locally (or the inability to visit them due to lockdown rules), service providers and doctors agreed that not enough specialised services were offered in any case (St Mungo’s 2011). Our research (mirroring that of others (Grassi, Caruso, and Costantini 2015; Skirbekk *et al*. 2011; Ruberton *et al*. 2016)) also shows the importance of establishing and maintaining healthy routines and habits (often connected to a continuity of care), as they can provide both stability and a sense of security for many. When people were no longer able to engage in their usual daily practices, we saw negative effects on both physical and mental health. This observation thus calls for a harm reduction approach to drug use; harm reduction emphasises the inevitability of drug and substance use in society and aims to ‘reduce the adverse health, social and economic consequences of the use of legal and illegal psychoactive drugs without necessarily reducing drug consumption’ (International Harm Reduction Association 2010). We believe there should be more conversation around embracing the approach of safe injection facilities in the UK (Safe Injection Sites: Coronavirus Underlines Why They Make Sense: Cato Institute), which would have benefited both institutions and individuals alike over the last 2 years.
2. *Incorporate, encourage and normalise the use of digital services*. The difficulties with the uptake of telemedicine we observed, might largely be a product of novelty and the fast and unprofessional set-up. With time and normalisation, people will adjust and some might perhaps even come to prefer it (as we also saw during our observations). Young adults have particularly benefited from mobile phone outreach and support; in some contexts, it can be used to provide community-based buprenorphine therapy for people experiencing homelessness (Glover *et al*. 2019; Harris *et al*. 2020). It can *increase* and further personalised care. The great potential of digital services, however, is contingent on several conditions: for example, access to a private and safe space for consultations (particularly important to make women experiencing homelessness feel safe), adequate connection to the internet and the availability of the necessary hardware and technology support and training (Thiyagarajan *et al*. 2020; Blandford *et al*. 2020). Without sufficient and necessary preparation and intention, it would not be a surprise if the incorporation of digital services only worsened situations, that is, disorganised, non-transparent, convoluted instructions would agitate, rather than alleviate problems.
  As such, the service design must be inclusive, equitable and comprehensive. For example, there must be different ways for people experiencing homelessness to set up video or phone consultation appointments (either people can directly call healthcare providers or support workers on their phones, or with provided phones and computers at centres or other local organisations). There must be an assurance of the continuity of care, where people experiencing homelessness would be able to access the same resource/person each time, from phones/video call options to the same healthcare providers. Furthermore, many experienced service providers have never used digital and online platforms. Their expertise is based on a very different and valuable skill set grounded in inperson human connection. Their knowledge must be consulted to make sure digitisation aids, rather than detracts, from their ability to work successfully with others. Comprehensive service design thus must also include communication and training with all staff in a coordinated way before roll-out. This will also require an accessible method of collecting feedback, allowing the various constituents of the set-up to feel comfortable to give suggestions for areas of improvement.
3. *Working towards a collaborative and integrated system of care*. Proper integration requires more than just the creation of a network of linkages (eg, through a unified software) between different actors (from people experiencing homelessness to specialised providers and the healthcare system). If the various actors are simply connected, with no attention paid to particular needs and backgrounds of various individuals, improvement would be uncertain. The disorganisation that came from the initial scramble to transition to lockdown rules would prolong and cause further aversion to digital services. This would not only discourage many from engaging, but it would also cause isolation—for all actors involved. To combat this, then, there must be a change towards intentional and integrated care provisions on a systematic level.

Overall, we continue to be surprised that the COVID-19 pandemic has provided substantial short- and mid-term alleviations for many people experiencing homelessness and shown us avenues and possibilities for longer-term change. Novel and unexpected policies have pushed many previously slowly developing approaches—especially around telemedicine—into a much quicker and wider experimental adoption. We sincerely hope that the health and housing fields will take seriously the learnings the pandemic has accelerated and given us, at overall great costs. Only then will we move substantially down the path of finding long-term and inclusive solutions that centre and uplift the people that have historically and structurally been most left excluded. However, these solutions will only come into fruition if they are supported by funding from long-term national policies that extend universal basic services to include individuals experiencing homelessness and those with complex care needs *in their complexity*.

## Data Availability

Data are available upon reasonable request.

## Notes

1. Obviously, the project was a short-term experiment; training would be easy to facilitate in a longer instalment of a similar service.
